# Time to revise our dialogue**:** how *flat* is the paediatric *flatfoot*?

**DOI:** 10.1186/s13047-017-0233-2

**Published:** 2017-11-21

**Authors:** Stewart C. Morrison, Juliet McClymont, Carina Price, Chris Nester

**Affiliations:** 1 School of Health Sciences, University of Brighton, 49 Darley Road, Eastbourne Campus, Brighton, BN20 7UR UK; 2 Centre for Health Research, University of Salford, Salford, UK

## Abstract

A recent systematic review of measures of foot development used the medial longitudinal arch profile as its primary indicator of development. A comparative analysis of existing studies was undertaken. This work confirmed changes with arch profile were age-dependent, although the age at which foot development ceased remains unknown. This work also highlighted the abundance of clinical measures used in existing research and outlined the challenges with drawing consensus from available data. There is a clear need to move this debate forward and, to do so, it is essential that scientific and clinical communities unite. It is time to abandon ill-defined measures of foot position, look beyond the medial longitudinal arch as a sole parameter of foot development and re-focus our perspective(s) on the paediatric foot in order to make advances with clinical practice and research.

We welcome the recent publication by Uden el al. [1] exploring characteristics of the typically developing paediatric foot. The shape, structure and function of the foot changes across infancy, childhood and adolescence and, as the authors state, this often poses challenges for clinicians confronted with the paediatric patient. For many years we have debated when to intervene with management of “*flatfoot*” and despite advances with our knowledge base, myths about the paediatric foot prevail. There is a stigma about the paediatric foot which extends beyond the podiatry profession and poses a barrier to our progress. Understanding the typical trajectory of the foot is essential but it is critical that we avoid pathologising typical foot development. This is harmful to our patients, perpetuates unnecessary expectation and damaging to our profession. We propose that it is time to revisit our dialogue and move away from attaching ill-defined labels (e.g. “*paediatric flatfoot*”, “*developmental flatfoot*”) to typical development.

We challenge the idea that the primary issue is how “*flat*” the foot is and even whether it is meaningful to call it “*flat*”. Evidence shows that foot shape is characterised by age-specific anatomical and aetiological factors [2, 3] yet, most clinical concerns are physiological, non-pathological and not requiring intervention [4]. Clarity emerging from robust, scientific data which quantifies the developmental trajectory of foot is needed and will be critical to resetting our theoretical, clinical and scientific perspectives about paediatric foot development.

It seems that the current approach to clinical practice is a distillation of the paradigms for managing adult feet, whereby some foot types are assumed problematic, even though there is no strong evidence for this. Throughout the Uden et al. [1] paper, and in much other literature, “*flatfoot*” is a strongly targeted foot type, often ‘diagnosed’ using one or more of the plethora of non-validated assessments. The lack of clinimetric data for these measures is a significant issue and, as the authors assert, children’s feet are developing structures and the absence of an arch is a typical stage of development. The developing foot is not structurally ‘flat’, it is a highly compliant, plastic and developing structure which responds to multiple determinants, many of which we do not understand. Recent work [3] used Magnetic Resonance Imaging to evaluate sub-talar morphology and demonstrated a relationship between the absence of the anterior sub-talar joint facet and development of the arch complex. This work highlights the need to constantly challenge the foundations of our existing knowledge and ensure clinical concepts are driven by research outcomes.

This work [3] helps point to another problem. Given the complexity and high intra-and inter-population variability of biological characteristics, measurement of these (i.e. the medial longitudinal arch) in isolation is flawed and focusing on “*flatfoot*” is futile unless it can be integrated within a wider developmental framework. It is time to look beyond the medial longitudinal arch and instead advance our understanding of the three-dimensional complexities of growth, morphology, anthropometric and functional norms of the paediatric foot. Our understanding of the three-dimensional function of the paediatric foot is emerging [5, 6] and technological advances must be embraced. There is no case for unreliable, uni-dimensional, static measurements.

Finally, it is important that we have rigorous data for health surveillance. A greater understanding of the clinical impact of variation in foot development, including how these may or may not relate to other markers of developmental issues (e.g. physical and cognitive), is required. Age related trajectories through developmental stages will ensure foot development can be measured throughout childhood and, as with head girth, height etc., measured against population norms. Even here however, it is not clear that feet developing towards the extremes of typical are anything other than examples of the normal statistical variation which is expected within a normally distributed population.

We don’t debate the importance of understanding foot development and this work [1] makes a useful addition to literature. But, we should ensure that we use our knowledge in a manner that underpins an ethical and reasoned approach to clinical practice. Our stance should be less about debating how flat the developing foot is and instead seek to understand more about the functional development of the foot for each child, and benchmark development against population norms. It is also fundamental that we re-frame the paradigms underpinning paediatric foot-care to help dispel unproven myths about children’s feet and strive to improve evidence based public health messages and thereby offer a more evidence informed approach to practice.
